# Protective activity of *Hertia cheirifolia* extracts against DNA damage, lipid peroxidation and protein oxidation

**DOI:** 10.1080/13880209.2016.1261907

**Published:** 2016-12-07

**Authors:** Seoussen Kada, Hamama Bouriche, Abderrahmane Senator, Ibrahim Demirtaş, Tevfik Özen, Bircan Çeken Toptanci, Göksel Kızıl, Murat Kızıl

**Affiliations:** a Laboratory of Applied Biochemistry, Faculty of SNV, University Ferhat Abbas, Sétif, Algeria;; b Department of Chemistry, Faculty of Science, Cankırı Karatekin University, Cankırı, Turkey;; c Department of Chemistry, Faculty of Science and Arts, Ondokuz Mayis University, Samsun, Turkey;; d Department of Chemistry, Faculty of Science and Arts, University of Dicle, Diyarbakır, Turkey

**Keywords:** Antioxidant activity, biomolecule lesions, medicinal plant, oxidative stress, polyphenols

## Abstract

**Context:** *Hertia cheirifolia* L. (Asteraceae), a perennial shrub widely distributed in Northern Africa, is traditionally used to treat inflammatory disorders.

**Objective:** The protective effect of methanol (Met E) and aqueous (Aq E) extracts of *Hertia cheirifolia* against DNA, lipid and protein oxidation was investigated.

**Materials and methods:** Different concentrations (50–1000 μg/mL) of *Hertia cheirifolia* aerial part extracts were examined against DNA, lipid and protein oxidation induced by H_2_O_2 _+ UV, FeSO_4_, and Fe^3+^/H_2_O_2_-ascorbic acid, respectively. The DPPH^•^, metal ion chelating, reducing power and β-carotene bleaching tests were conducted.

**Results:** Both extracts were rich in polyphenols, flavonoids and tannins, and were able to scavenge DPPH^•^ with IC_50_ values of 138 and 197 μg/mL, respectively. At 300 μg/mL, Aq E exerted stronger chelating effect (99%) than Met E (69%). However, Met E reducing power (IC_50 _=_ _61 μg/mL) was more than that of Aq E (IC_50 _=_ _193 μg/mL). Both extracts protected from β-carotene bleaching by 74% and 94%, respectively, and inhibited linoleic acid peroxidation. The inhibitory activity of Aq E extract (64%) was twice more than that of Met E (32%). Interestingly, both extracts protected DNA against the cleavage by about 96–98%. At 1 mg/mL, Met E and Aq E restored protein band intensity by 94–99%.

**Conclusions:** *Hertia cheirifolia* exhibits potent antioxidant activity and protects biomolecules against oxidative damage; hence, it may serve as potential source of natural antioxidant for pharmaceutical applications and food preservation. This is the first report on the protective activity of this plant against biomolecule oxidation.

## Introduction

Exogenous chemicals, physical sources and endogenous metabolic processes in the human body might produce highly reactive oxygen species (ROS). The excess of uncontrolled ROS production leads to oxidative stress triggering damage in the cell, leading to cell damage and homeostatic disruption, and so forth, ultimately resulting in a number of human diseases. Lipids, DNA and proteins are the major targets of ROS in the body (Lobo et al. 2010).

At the cellular level, ROS oxidize lipids to generate peroxides and aldehydes. The increased formation of these products has been observed in atherosclerosis, ischemia-reperfusion, heart failure, Alzheimer’s disease, rheumatic arthritis, cancer, and other immunological disorders (Ramana et al. 2013). Moreover, ROS are the main cause of deterioration of many foods, leading to the formation of toxic compounds and minimizing the nutritional value of foods.

DNA is a target for excess oxidative stress, which attacks the bases and sugar moieties, creating strand breaks, altered gene expression, and ultimately mutagenesis (Sharma et al. 2012). Oxidative DNA damaged has been thought to be a critical contributor to the development of aging and some degenerative diseases (Chao et al. 2013). Moreover, continuous oxidative damage to DNA is believed to be a significant contributor to the development of many cancers (Broustas & Lieberman 2014).

Proteins are also exposed to ROS attacks, which may cause modulation of their activity through nitrosylation, carbonylation, disulfide bond formation, and glutathionylation (Sharma et al. 2012). Furthermore, as a consequence of excessive ROS production, site-specific amino acid modification, fragmentation of the peptide chain, aggregation of cross-linked reaction products, altered electric charge and increased susceptibility of proteins to proteolysis (Moller & Kristensen 2004). These modifications lead to functional changes that disturb cellular metabolism. The accumulation and damaging actions of oxidized proteins was observed in several pathological states such as diabetes, neurodegenerative diseases and aging (Pandey et al. 2010; Chen et al. 2012; Rahman et al. 2012).

The development and the utilization of more effective antioxidants of natural origin, which have a higher bioavailability and therefore, higher protective efficacy than synthetic antioxidants are desired. Generally, natural antioxidants from the plant kingdom have been identified as major health beneficial compounds, and medicinal plants are considered as natural sources for alternative medicines. Antioxidants such as β-carotene play a vital role in the prevention of various cardiovascular diseases and cancer (Lobo et al. 2010).

*Hertia cheirifolia* L. (Asteraceae) is perennial shrub found throughout the Mediterranean area. It is widely distributed in Northern Africa (Beniston & Beniston 1984), and known for its uses in the indigenous medicine for a variety of purposes. It is used by traditional healers for the treatment of spasm, inflammation, diarrhea and hemorrhoid (Iserin 2001). The phytochemical analysis of this plant showed the presence of sesquiterpenoids and steroids (Aclinou et al. 1991; Ammar et al. 2009). However, few studies on biological activities of *H. cheirifolia* have been reported. Therefore, the current study evaluates the antioxidant potency and the protective activity of *H. cheirifolia* methanol and aqueous extracts against biomolecule oxidative damages.

## Materials and methods

### Plant material

The aerial parts of *Hertia cheirifolia* were collected in June 2010 from Setif, in eastern Algeria. The plant was identified and authenticated taxonomically by Dr. N. Boulaacheb, Univesity of Sétif 1, Algeria. A voucher specimen (No. H.C. 2010–1) was preserved at the local Herbarium of Botany, Department of Botany, University of Sétif 1, Algeria. Leaves were air-dried at room temperature and then reduced to powder.

### Preparation of plant extracts

Methanol extract (Met E) of *H. cheirifolia* leaves was prepared as described previously (Bouriche et al. 2016). Briefly, plant dried leaves (50 g) were pulverized and macerated twice with 500 mL of methanol 80% and then with 50% methanol. After filtering, the filtrate was concentrated under reduced pressure at 40 °C. The residue was lyophilized to give a brown powder (yield: 19%) and then stored at −32 °C until use.

Aqueous extract (Aq E) was prepared according to the traditional method by boiling 50 g of powdered plant in 500 mL of distilled water for 20 min. After filtration, the filtrate was lyophilized to give a brown powder (yield: 17%).

### Polyhenol, flavonoid and tannin determination

Total polyphenolic content was determined according to Li et al. (2007); gallic acid (10–180 μg/mL) was used as the standard. Samples of 40 μL of extract solution (1 mg*/*mL) were mixed with 200 μL Folin–Ciocalteau’s phenol reagent 10% in water. After 4 min of incubation, 0.4 mL of 20% Na_2_CO_3_ was added. The reaction tubes were further incubated for 2 h at room temperature and the absorbance was measured at 760 nm. The concentration of total phenolic compounds in the extract was determined as mg of gallic acid equivalents per g of extract (GAE/g extract).

Total flavonoid content was quantified according to Bahorun et al. (1996) using quercetin (2–20 μg/mL) as standard. Briefly, samples of 1 mL of extract solution (1 mg/mL) were incubated in the presence of 1 mL of AlCl_3_ (2%) for 10 min at room temperature. The absorbance was measured at 430 nm. Total flavonoid content was expressed as mg quercetin equivalent per g of extract (QE/g extract).

Tannin content was determined using the hemoglobin precipitation assay according to Bate-Smith (1973), using tannic acid (100–550 μg/ml) as standard. A volume of 450 mL of distilled water was added to 5 mL of bovine blood (obtained from the slaughterhouse) to reach 1.6 of absorbance at 578 nm. An aliquot of 0.5 mL of each extract was mixed with 0.5 mL of hemolysis bovine blood to reach a final concentration of 1 mg/mL, then the mixture was centrifuged at 480*g* for 20 min and the absorbance was measured at 578 nm. Tannin content was expressed as mg tannic acid equivalent per g of extract (TAE/g extract).

### Free radical scavenging activity

The free radical scavenging activity of the extracts was measured according to the method of Que et al. (2006). The solution of the free DPPH^•^ in ethanol (0.1 mM) was prepared and 0.5 mL of aqueous or methanol extracts at different concentrations (10–400 μg/mL) were added. The mixture was shaken vigorously and left standing at room temperature for 30 min. After the incubation, the absorbance of the resulting solution was measured at 517 nm. Butylated hydroxytoluene (BHT, 10-400 μg/mL) was used as standard antioxidant. The ability to scavenge the DPPH˙ was calculated using the following equation:

$${\mathrm{DPPH}}^{•}\;\mathrm{scavenging}\;\mathrm{activity}\;(\%)=[(A_{0}-A_{1})/A_{0}]\times 100$$

where *A*_0_ is the absorbance of the control, and *A*_1_ is the absorbance of the sample.

Regression equations for defining IC_50_ values of Met E, Aq E and BHT were: *y* = 0.362*x* + 0.044, *y* = 0.229*x* + 4.852 and *y* = 1.117*x* + 0.447, respectively.

### Ferrous ions chelating activity

The ferrous ion chelating activity of methanol and aqueous extracts was estimated by the method of Le et al. (2007). Briefly, 700 μL of the extracts samples at different concentrations (20–300 μg/mL) were added to a solution of 0.6 mmol/L FeCl_2_ (50 μL). The reaction was initiated by the addition 50 μL of ferrozine (5 mM) and the mixture was shaken vigorously and left standing at room temperature for 10 min. The absorbance of the solution was then measured at 562 nm. EDTA (2-300 μg/mL) was used as a reference. The percentage of inhibition of ferrozine–Fe^2+ ^complex formation was calculated using the following formula:

$$\mathrm{Ferrous}\;\mathrm{ions}\;\mathrm{chelating}\;\mathrm{activity}\;\left(\%\right)=[(A_{0}-A_{1})/A_{0}]\times 100$$

where *A*_0_ is the absorbance of the control (control contained FeCl_2_ and ferrozine; complex formation molecules), and *A*_1_ is the absorbance of the sample.

Regression equations for defining IC_50_ values of Met E, Aq E and EDTA were: *y* = 0. 299 *x* + 0.909, *y* = 0.815 *x* + 0.071 and *y* = 8.405*x* − 0.197, respectively.

### Reducing power

The reducing power of the extracts was determined according to Oyaizu (1986). Each extract (20–350 μg/mL) in 2.5 mL of distilled water was mixed with 2.5 mL of 200 mM sodium phosphate buffer (pH 6.6) and 2.5 mL of 1% potassium ferricyanide. The mixture was incubated at 50 °C for 20 min. Then, 2.5 mL of 10% TCA were added and the mixture was centrifuged at 200*g* for 10 min. The upper layer (2.5 mL) was mixed with 2.5 mL of deionized water and 0.5 mL of 0.1% FeCl_3_. BHT (5–30 μg/mL) was used as a standard antioxidant. The absorbance was measured at 700 nm. Higher absorbance indicates higher reducing power. Equations for defining IC_50_ values of Met E, Aq E and BHT were: *y* = 0.009*x* − 0.049, *y* = 0.0022 *x* +0.0754 and *y* = 0.042*x* − 0.222, respectively.

### β-Carotene bleaching method

The antioxidant activity of the extracts was determined according to the β-carotene bleaching method described by Tepe et al. (2006). A reagent mixture containing 1 mL of β-carotene solution (0.2 mg/mL in chloroform), 25 μL of linoleic acid and 200 μg of Tween 40. After removing the chloroform by using a rotary evaporator (Buchi), 100 mL of oxygenated distilled water was added. The mixture was stirred vigorously to form a liposome solution. Aliquots (5 mL) of the liposome solution were transferred to a series of test tubes containing 200 μL of extract (2 mg/mL), 200 μL of BHT (2 mg/mL) or 200 μL distilled water (control). The absorbance was measured immediately (*t* = 0 min) at 490 nm using a spectrophotometer (Hitachi U 2000, Tokyo, Japan). Subsequently, the reaction mixtures were incubated at 50 °C. The absorbance was measured again at time intervals of 15 min for 2 h (*t* = 120 min). All samples were assayed in triplicate. BHT was used as standard antioxidant. A second emulsion consisting of 100 mL distilled water, 25 μL of linoleic acid and 200 mg of Tween 40 was also prepared. Distilled water (200 μL) with 5 mL of this second emulsion was used to zero the spectrophotometer. The rate of β-carotene bleaching (*R*) was calculated according to the following equation: *R* = ln (*A*_o_/*A_t_)/t* where ln is the natural logarithm, *A*_0_ is absorbance at time 0, *A_t_* is absorbance at time *t*, and *t* is 15, 30, 45, 60, 75, 90, 105 or 120 min. The antioxidant activity (%) was calculated in terms of percentage inhibition relative to the control, using the following equation:

$$\mathrm{Antioxidant}\;\mathrm{activity}\;\left(\%\right)=[(R_{\mathrm{control}}-R_{\mathrm{sample}})/R_{\mathrm{control}}]\times 100$$

### Lipid peroxidation assay

The anti-peroxidation activity of the extracts was performed according to a modified method of Choi et al. (2002). This method was developed for the measurement of lipid peroxidation, with linoleic acid as the source of lipid in an oxidation system catalyzed by Fe-ascorbate. Samples of extracts (50–500 μg/mL) were mixed with linoleic acid solution (0.28 mg linoleic acid and 0.28 mg Tween 20 in 500 mL of 100 μM phosphate buffer (pH 7.4) and 150 μL of 10 μM ascorbic acid solution. The mixture was vortexed and sonicated to obtain a homogeneous emulsion solution. The linoleic acid peroxidation was initiated by the addition of 0.1 mL FeSO_4_ (10 μM) and incubation at 37 °C for 60 min. The mixture was cooled and 1.5 mL of TCA (10% in 0.5% HCl) was added. Then, 3 mL TBA (1%, in 50 mM NaOH) was added and the mixture was heated in a water bath at 90 °C for 60 min. After cooling, aliquots of 2 mL were taken from each sample and vortexed with 2 mL of butanol and centrifuged at 1000*g* for 30 min. The upper layer solution was separated for the pectrophotometric measurement. The absorbance of each solution at 532 nm was recorded and the percentage of linoleic acid peroxidation inhibition was defined according the following equation:

$$\mathrm{Linoleic}\;\mathrm{acid}\;\mathrm{peroxidation}\;\mathrm{inhibition}\;\mathrm{activity}\;\left(\%\right)=[(A_{o}-A_{1})/A_{o}]\times 100$$

where *A*_o_ is the absorbance of control reaction (containing all reagents except the extracts) and *A*_1_ is the absorbance of the sample with the extracts or the standard.

### DNA strand scission assay

The protective activity of methanol and aqueous extracts of *H. cheirifolia* against DNA damage was checked on pBluescript M13 + plasmid DNA (Stratagene, La Jolla, CA). Plasmid DNA was isolated by Qiagene plasmid miniprep kit, then oxidized with H_2_O_2 _+ UV treatment in the presence or absence of different concentrations (100, 250, 350 and 500 μg/mL) of methanol or aqueous extracts of *H. cheirifolia*, and checked on 1% agarose according to a modified method of Attaguile et al. (2000). The experiments were performed in a volume of 10 μL in a microcentrifuge tube containing 200 ng of plasmid DNA in phosphate buffer (7.14 mmol phosphate and 14.29 mmol NaCl, pH 7.4) and H_2_O_2_ was added at a final concentration of 2.5 mmol*/*L with and without 1 μL of methanol and aqueous extracts. The reactions were initiated by UV irradiation and continued for 5 min on the surface of a UV transilluminator with intensity 8000 μW/cm^2^ at 300 nm under room temperature. After irradiation, the reaction mixture (10 μL) with gel loading dye was placed on 1% agarose gel for electrophoresis. Electrophoresis was performed at 40 V for 3 h in the presence of ethidium bromide (10 mg*/*mL). Untreated pBluescript M13 + plasmid DNA was used as a control in each run of gel electrophoresis along with partial treatment (i.e. only UV treatment and only H_2_O_2_). Percent inhibition of the DNA strand scission was calculated using the following equation:

Inhibition (%)=I-(Sm+a-Sc)/(Sm-Sc)

where Sm + a is the percentage remaining supercoiled DNA after treatment with UV + H_2_O_2_ in the presence of the extracts, Sc is percentage remaining supercoiled DNA in the control untreated plasmid and Sm is percentage remaining supercoiled DNA with UV + H_2_O_2_ without extracts.

Densitometry analysis of treated and untreated pBluescript M13 + plasmid DNA gel was scanned using Gel Documentation System (Gel-Doc-XR; BioRad, Hercules, CA). Bands on the gels were quantified by discovery series Quantity One program (version 4.5.2, BioRad, Hercules, CA).

### Protein oxidation assay

The protective ability of methanol and aqueous extracts of *H. cheirifolia* against H_2_O_2_/Fe^3+^/ascorbic acid protein attack was investigated as described by Kizil et al. (2011). BSA (1 mg/mL), used as a model protein, was dissolved in 20 mM potassium phosphate buffer (pH 7.4) and then 50 μM FeCl_3_, 1 mM H_2_O_2_ and 100 μM ascorbic acid were added to the reaction mixture. This mixture was incubated in the presence or absence of methanol and aqueous extracts of *H. cheirifolia* at different concentration (50–1000 μg/mL) in a final volume of 1.2 mL. After incubation for 3 h at 37 °C, the reaction mixture was analyzed by electrophoresis in 10% SDS polyacrylamide gel (Laemmli 1970). Samples were mixed with equal volumes of sample buffer (Tris HCl pH 6.8, 2% SDS, 5% 2-mercaptoethanol, 10% sucrose, and 0.002% bromophenol blue) and boiled for 5 min, and then 5 μL of each sample was electrophoresed by SDS-PAGE. The gel was run in a BioRad tank in running buffer (25 mM Tris pH 8.3, 190 mM glycine, and 0.1% SDS) at a maximum voltage and a constant current of 25 mAmp for a mini gel, using a BioRad 1000/500 power supply. Gels were stained with 0.15% Coomassie Brilliant Blue R-250 for 2 h and then distained and digitally photographed.

Protein band intensity was estimated using the Gel Documentation System (Gel-Doc-XR; BioRad, Hercules, CA) and standardized with respect to the control group. Bands on the gels were quantified by discovery series Quantity One program (version 4.5.2, BioRad Co.).

### Statistical analysis

Results are expressed as mean ± SD. The statistical analysis was performed using one way ANOVA. The differences were considered statistically significant at *p* < 0.05.

## Results

### Total polyphenol, flavonoid and tannin content

Results showed that methanol extract of *H. cheirifolia* contains the highest amount of polyphenols and tannins compared to aqueous extract. However, both extracts contain the same quantity of flavonoids (Table 1).

**Table 1:** Polyhenol, flavonoid and tannin content in methanol extract (Met E) and aqueous extract (Aq E) of *H. cheirifolia*.

Extract	Polyphenols (mg GAE/g dried extract)	Flavonoids (mg QE/g dried extract)	Tannins (mg TAE/g dried extract)
Met	89 ± 0.00	4 ± 0.02	79 ± 0.03
Aq	54 ± 0.01	3 ± 0.00	11 ± 0.01

Values are mean of triplicate determination (*n* = 3) ± SD.

### Free radical scavenging activity

**Figure 1.**
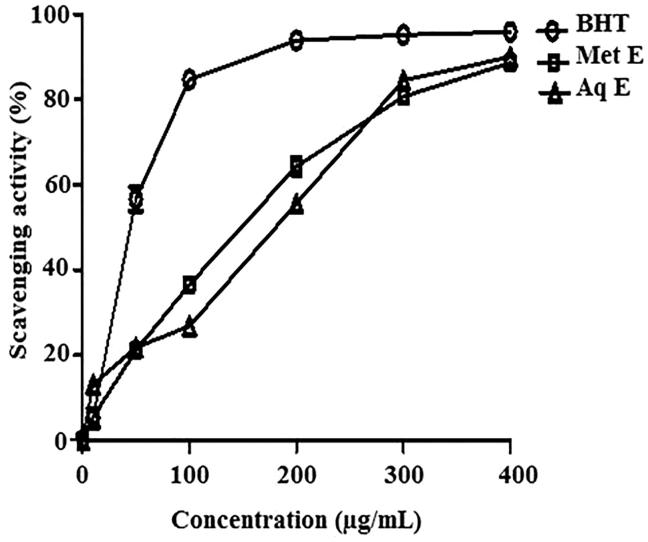
Free radical scavenging activity of methanol extract (Met E), aqueous extract (Aq E) of *H. cheirifolia* and BHT. Values are means ± SD (*n* = 3).

Methanol and aqueous extracts of *H. cheirifolia* showed a concentration-dependent scavenging activity of DPPH^•^. However, methanol extract was more active (IC_50 _=_ _138 μg/mL) than aqueous extract (IC_50 _=_ _197 μg/mL). This activity was less than that obtained with BHT (IC_50 _=_ _44.36 μg/mL), used as a standard antioxidant (Figure 1).

### Ferrous ions chelating activity

**Figure 2.**
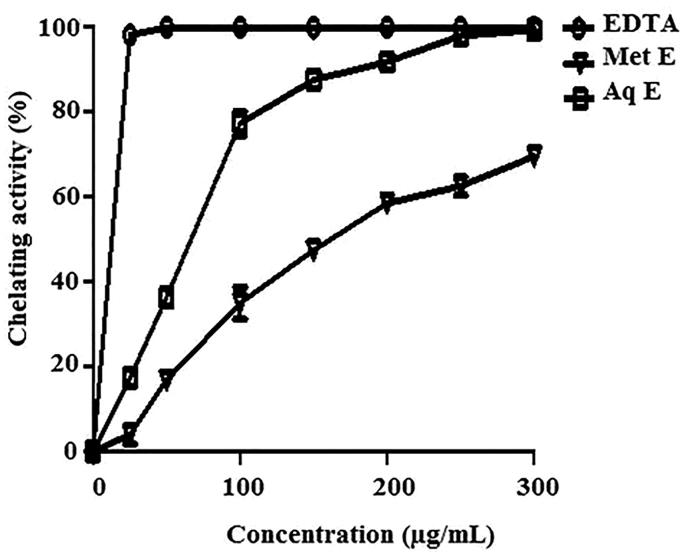
Ferrous ion chelating activity of methanol (Met E), aqueous extract (Aq E) of *H. cheirifolia* and the standard EDTA. Values are expressed as means ± SD (*n* = 3).

Both extracts of *H. cheirifolia* were able to chelate ferrous ions in a concentration-dependent manner. At 300 μg/mL, *H. cheirifolia* Aq E exerted a strongest chelating effect (99%) with an IC_50_ value of 61 μg/mL followed by *H. cheirifolia* Met E (69%) with an IC_50_ value of 170 μg/mL. This activity was less important than that obtained with the standard chelator EDTA (IC_50 _=_ _5.97 μg/mL) (Figure 2).

### Reducing capacity

**Figure 3.**
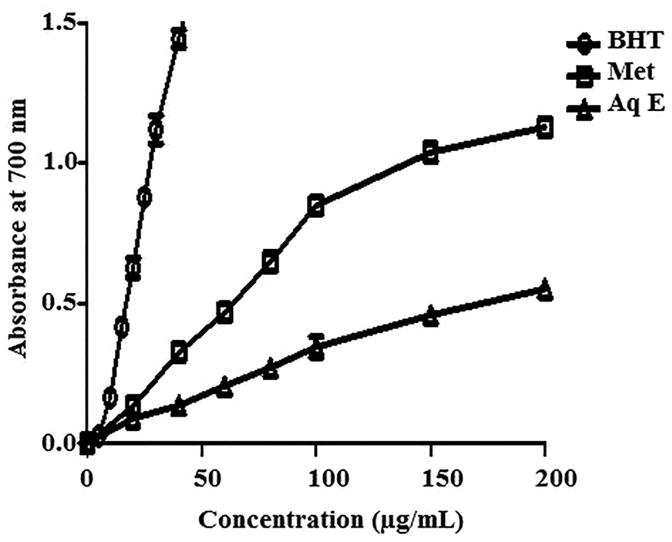
Reducing power capacity of methanol extract (Met E), aqueous extract (Aq E) of *H. cheirifolia* and BHT. Values are expressed as means ± SD (*n* = 3).

The reductive capability of *H. cheirifolia* extracts compared with BHT is illustrated in Figure 3. Methanol extract exerted a strong reducing power (IC_50 _=_ _61 μg/mL) compared with the Aq E (IC_50 _=_ _193 μg/mL). However, this reductive capability is less important than that observed with BHT (IC_50 _=_ _17 μg/mL).

### β-Carotene bleaching

**Figure 4.**
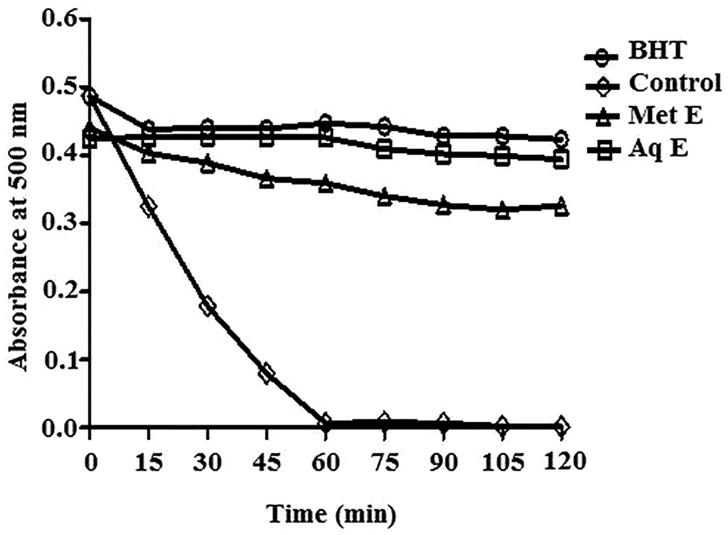
Kinetics of antioxidant activity of *H. cheirifolia* aqueous extract (Aq E), methanol extract (Met E) and the standard antioxidant (BHT) in β-carotene-linoleic acid system. Values are expressed as means ± SD (*n* = 3).

The changes in the absorbance under the influence of *H. cheirifolia* methanol and aqueous extracts compared to BHT during 120 min are shown in Figure 4. As shown in this figure, in the presence of both extracts of *H. cheirifolia*, the absorbance was very low and remained stable during all the incubation time. The inhibition of β-carotene bleaching exerted by 2 mg/mL of methanol and aqueous extracts of *H. cheirifolia* were about 74% and 94%, respectively. The activity of aqueous extract is very close to that of BHT (92%), while the activity of methanol extract is less than that of BHT used as a standard antioxidant.

### Protective effect of *H. cheirifolia* extracts on lipid peroxidation

**Figure 5.**
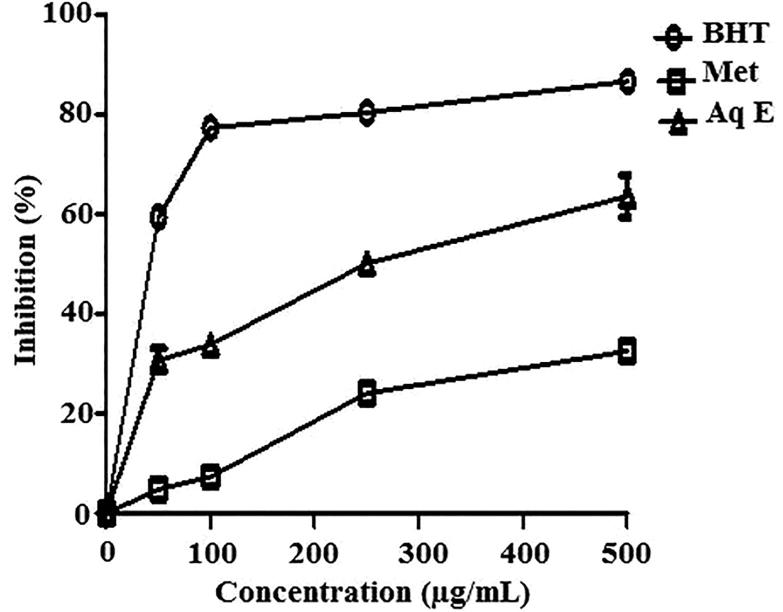
Inhibition of lipid peroxidation by methanol extract (Met E), aqueous extract (Aq E) of *H. cheirifolia* and standard (BHT). Values are expressed as means ± SD (*n* = 3).

Both extracts of *H. cheirifolia* showed a significant inhibition of linoleic acid peroxidation. At 500 μg/mL, *H. cheirifolia* Aq E was the most efficient with a percentage inhibition value of 64%, while *H. cheirifolia* Met E exerted only 32% of inhibition. This inhibition was less effective than that observed with BHT (86.65%), at the same concentration (Figure 5).

### Protective effect of *H. cheirifolia* extracts on DNA damage

**Figure 6.**
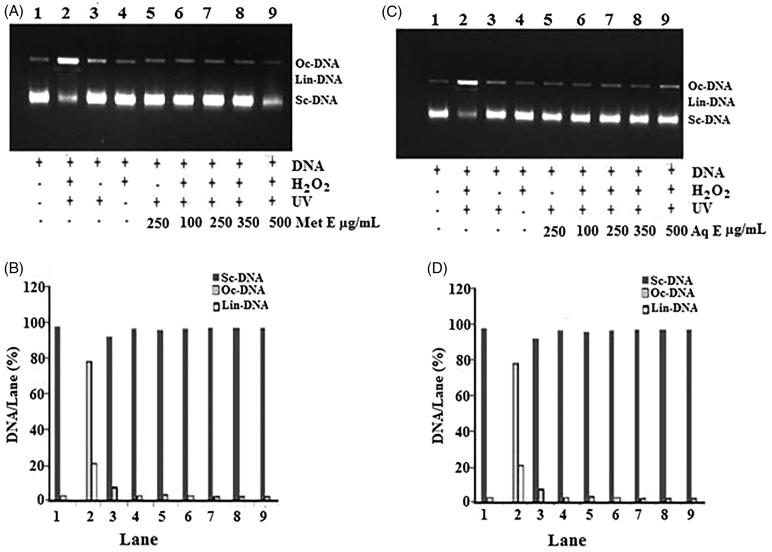
DNA damage protective activity of *H. cheirfolia* Met E (A and B) and Aq E (C and D). A and C: Electrophoretic pattern of pBluescript M13 + DNA after UV-photolysis of H_2_O_2_. B and D: the quantified band intensity for the scDNA, ocDNA and linDNA quantified by discovery series Quantity One programme (version 4.5.2, BioRad Co.). Lane 1: untreated and non-irradiated DNA, Lane 2: DNA + H_2_O_2_ (2.5 mM) + UV, Lane 3: DNA + UV, Lane 4: DNA + H_2_O_2_ (2.5 mM), Lane 5: DNA + Met E or Aq E (250 μg/mL) + UV, Lane 6: DNA + Aq E or Met E (100 μg/mL) + H_2_O_2_ (2.5 mM) + UV, Lane 7: DNA + Aq E or Met E (250 μg/mL) + H_2_O_2_ (2.5 mM) + UV, Lane 8: DNA + Aq E or Met E (350 μg/mL) + H_2_O_2_ (2.5 mM) + UV, Lane 9: DNA + Aq E or Met E (500 μg/mL) + H_2_O_2_ (2.5 mM) + UV.

Electrophoretic pattern of DNA after UV-photolysis of H_2_O_2_ in the presence of different concentrations (100, 250, 350 and 500 μg/mL) of Met E and Aq E of *H. cheirifolia* is shown in Figure 6. The conversion of supercoiled circular (scDNA) to open circular form (ocDNA) derived from pBluescript M13 + DNA plasmid showed two bands on agarose gel electrophoresis (lane 1), the faster moving band corresponded to the native form of scDNA and the slower moving band was the ocDNA form. The UV irradiation of DNA in the presence of H_2_O_2_ (lane 2) resulted in the cleavage of scDNA to ocDNA form and linear form (linDNA). It was noted that only UV treatment (lane 3), only H_2_O_2_ treatment (lane 4) and only UV treatment with 250 of extract (lane 5) could not induce damage, as noted in combined treatment; UV + DNA + H_2_O_2_ (lane 2). The addition of the extracts (lanes 6–9) to this reaction mixture similarly induced a partial recovery of scDNA. In fact, at 100, 250, 350 and 500 μg/mL of Aq E, the intensity of scDNA bands scanned from the agarose gel electrophoretic patterns were 97%. Similarly, at the same concentrations of Met E, the intensity of scDNA bands were 96–98%, respectively, as compared with the DNA control (lane 2).

### Protective effect of *H. cheirifolia* extracts on protein oxidation

**Figure 7.**
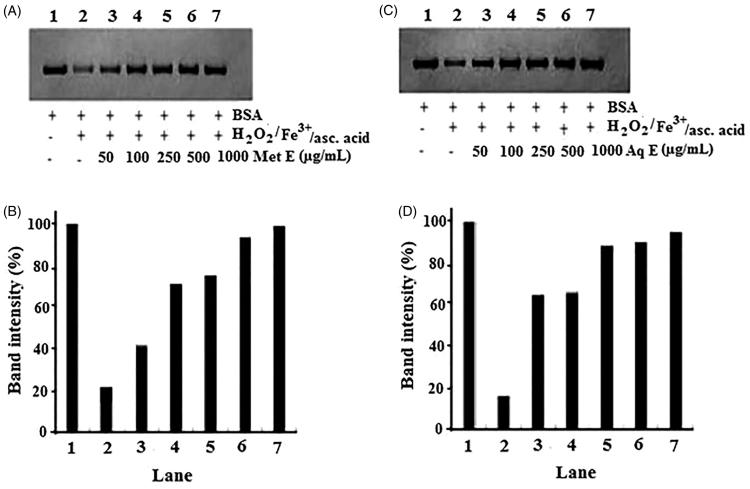
Protection of BSA oxidative damage by aqueous extract (Aq E; A and B) and methanol extract (Met E; C and D) of *H. cheirifolia*. BSA was oxidized by Fenton system (H_2_O_2_/Fe^3+^/ascorbic acid). The reaction mixture (1.2 mL) containing *H. cheirifolia* Met E or Aq E (50–1000 μg/mL), BSA (1 mg/mL), FeCl_3_ (50 μM), H_2_O_2_ (1 mM), ascorbic acid (100 μM) and potassium phosphate buffer (20 mM, pH 7.4) was incubated for 3 h at 37 °C. The oxidative damage of BSA was quantified by SDS-PAGE. SDS gels were digitally photographed and the integrated density of bands was measured using Discovery series Quantity One Program (version 4.5.2. BioRad Co.).

Electrophoretic patterns of BSA after incubation 3 h with Fe^3+^/H_2_O_2_/ascorbic acid system in the presence or absence of different concentrations of methanol and aqueous extracts of *H. cheirifolia*, and the corresponding densitometry analyses of the corresponding bands are presented in Figure 7(A,B). The density of BSA band of control (lane 2) decreased to about 16% and 23%, after 3 h of incubation with Fe^3+^/H_2_O_2_/ascorbic acid system. The treatment with different concentrations (50–1000 μg/mL) of methanol and aqueous extracts of *H. cheirifolia* (lanes 3–7) showed protective effect on BSA degradation induced by Fe^3+^/H_2_O_2_/ascorbic acid. This protective activity was concentration-dependent. Indeed, at 50, 100, 250, 500 and 1000 μg/mL, *H. cheirifolia* methanol extract restored the BSA band intensity by 41%, 71%, 75%, 93% and 99%, respectively, whereas *H. cheirifolia* aqueous extract restored the BSA band intensity by 64%, 65%, 88%, 90% and 94%, respectively, as compared to the control.

## Discussion

The use of natural antioxidant, that can suppress oxidative damage, can be beneficial in preventing diseases. Mechanisms of antioxidant action include suppressing of ROS formation, either by inhibition of enzymes or by chelating trace elements involved in free-radical production, scavenging reactive species and up-regulating or protecting antioxidant defences. In the present study, the free radical scavenging activity of aqueous and methanol extracts of *H. cheirifolia* was evaluated using DPPH^•^ test, which measures the capacity of the extracts to scavenge the stable free radical formed in solution, by donating of a hydrogen atom or an electron (Kedare & Singh 2011). Methanol and aqueous extracts of *H. cheirifolia* exerted concentration-dependent free radical scavenging activity; however, the methanol extract was more potent. This capacity is probably highly related to the phenolic content. Indeed, our results showed that the methanol extract of this plant contains higher polyphenols and flavonoids than aqueous extract. Phenolic compounds are generally more soluble in polar organic solvents than in water ones (Munro et al. 2015). Hence, the increased amount of these compounds in the methanol extract must have conferred it with a more potent antiradical property than the aqueous extract. Previous study also demonstrated that the methanol extract from the same plant had a strong ability to act as antiradical (Bousselsela et al. 2012). In addition, both extracts exhibited chelating capacity, and the activity of the aqueous extract was unexpectedly better than that of methanol extract; in perfect contrast to those found for free radical scavenging. The different phenolic components present in aqueous and methanolic extracts, as for example, flavonoids, may have contributed to these results. Indeed, it has been reported that some flavonoids, such as naringin, pelargonidin, phloridzin, and hesperitin had no chelating activity, contrary to apigenin, diosmin, phloretin, fisetin, cyanidanol, taxifolin, and naringenin, which presented good chelating properties (Van Acker et al. 1996). Furthermore, *H. cheirifolia* extracts exerted a concentration-dependent reducing activity. However, methanol extract showed a higher reducing activity than the aqueous extract. This reducing capacity is probably due to the presence of active components that act as reductants. It has been reported that electron donating capacity, reflecting the reducing power of phenolics and flavonoids, serve as a significant indicator of its potential antioxidant activity (Dai & Mumper 2010).

In β-carotene bleaching test, oxidation of linoleic acid generates free radicals, which attack the highly unsaturated β-carotene molecules to reacquire a hydrogen atom. During this reaction, the molecule of β-carotene loses its conjugation and as a consequence its orange colour disappears (bleaching) by oxidation (Duan et al. 2006). Both studied extracts can reduce the extent of β-carotene destruction by neutralizing free radicals formed in the system. As cited before, *H. cheirifolia* methanol and aqueous extracts possess a strong radical quenching activity. So, the extracts inhibited of linoleic acid peroxidation by extending the lag phase and reducing the propagation rate, thus reflecting typical characteristic of a chain-breaking antioxidant, similar as the standard antioxidant BHT. Lipid peroxidation is a chain reaction initiated by the hydrogen abstraction or the addition of an oxygen radical, resulting in the oxidative damage of polyunsaturated fatty acids (Yin et al. 2011). In order to confirm the protective effect *H. cherifolia* extracts against lipid peroxidation, another known test, linoleic acid catalyzed by Fe^2+^-ascorbate, has been used in this study. Ferrous (Fe^2+^) ions are the most powerful pro-oxidants among the various species of metal ions (Halliwell & Gutteridge 1984), and the transition of these metal ions can stimulate lipid peroxidation via Fenton reaction, and accelerate lipid peroxidation by decomposing lipid hydro peroxides into peroxyl and alkoxyl radicals that can propagate the chain of lipid peroxidation (Ayala et al. 2014). So, minimizing ferrous ions may afford protection against oxidative damage by inhibiting production of ROS and lipid peroxidation. In this system, *H. cheirifolia* extracts recorded potent lipid peroxidation inhibition. Both extracts may serve as secondary antioxidants, as they reduce the redox potential and thereby stabilizing the oxidized form of the ferrous ions. The ability of the extracts to scavenge radicals and then inhibit lipid peroxidation may be attributed, as cited before, to their constituents that are electron donors, which can react with free radicals to convert them to more stable products. Indeed, *H. cheirifolia* methanol extract is rich in phenolic compounds (Bouriche et al. 2016). These compounds are potent antioxidants and inhibit strongly the lipid peroxidation (Lizcano et al. 2012; Saleh et al. 2015).

The use of the antioxidants to prevent UV-induced DNA damage has aroused considerable interest because of their potential beneficial effects on human health in fighting diseases. In the current study, the DNA cleavage analysis demonstrated the strong DNA protective activity of *H. cheirifolia* extracts. The generation of DNA oxidative damage is hypothesized to occur via the production of ROS (Moller et al. 2014). The UV irradiation of DNA in the presence of H_2_O_2_ caused the cleavage of super coiled DNA to open circular and further to linear forms, indicating that hydroxyl radical (OH**^•^**) generated by UV photolysis of H_2_O_2_ induced DNA strand scission and breakage. The hydroxyl radical is known to react with all components of DNA such as purine and pyrimidine bases as well as the deoxyribose backbone (Cadet et al. 2014). Both the extracts of *H. cheirifolia* protected pBluescript M13 + super coiled double-strand DNA from hydroxyl radical-induced strand scission. In the presence of an increasing concentration of these extracts, the proportion of both ocDNA and linDNA decreased significantly, while the amount of the residual super coiled DNA was recovered. Accordingly, it should be taken into consideration that the DNA protecting ability is related to the antioxidant capacity of the extracts, which may stabilize the DNA damage by neutralizing or destroying the free radicals. This activity could be assigned to the presence of bioactive compounds. In fact, the studied extracts, in particular methanol extract is rich in rutin and phenolic acids like *p*-coumaric acid, ferulic acid and cinnamic acid (Bouriche et al. 2016). Several phenolic acids protect DNA against the mutagenic and toxic effects of UV and H_2_O_2_ (Sevgi et al. 2015). Moreover, phenolic acids and flavonoids can prevent the production of ROS by complexing cations such as copper and iron that participate in hydroxyl radical formation (Jun et al. 2007; Dai & Mumper 2010).

Proteins are also susceptible to oxidation by ROS. Several amino acids, especially arginine, histidine, methionine and cysteine tend to undergo oxidation under antioxidant deficiency conditions. Oxidative protein damage has been demonstrated to play a significant role in aging and several pathological events (Rahman et al. 2012). So, measurement of protein oxidation has been used as a sensitive assay to evaluate oxidative protein damage. In the current study, densitometric analysis of protein bands and quantified gel image showed the protective effect of *H. cheirifolia* extracts against ROS attacks. At 1 mg/mL, methanol and aqueous extracts of *H. cheirifolia* protected significantly BSA and restored highly the protein band intensity. This protective ability is mainly due the antioxidant activity of the extracts. In fact, phenolic compounds are considered as major active components of the plant extracts responsible for the strong antioxidant capacity (Wang et al. 2009; Zhao et al. 2014).

## Conclusion

*Hertia cheirifolia* extracts exhibit a good antioxidant activity and conferred protection against biomolecule oxidative damage. So, *H. cheirifolia* extracts could be a promising antioxidant source for the prevention and/or treatment of oxidative stress-related diseases or as additives in the foods, as it could retard oxidative degradation of protein and lipids and thereby improve the nutritive value of food.
